# The manufacturing process should remain the focus for severe febrile reactions in children administered an Australian inactivated influenza vaccine during 2010

**DOI:** 10.1111/irv.12337

**Published:** 2015-12-11

**Authors:** Jean Li‐Kim‐Moy, Robert Booy

**Affiliations:** ^1^ National Centre for Immunisation Research and SurveillanceThe Children's Hospital at WestmeadSydneyNSWAustralia; ^2^ Sydney Medical SchoolThe University of SydneySydneyNSWAustralia; ^3^ The Children's Hospital at WestmeadSydneyNSWAustralia; ^4^ Marie Bashir Institute for Infectious Diseases & BiosecurityUniversity of SydneySydneyNSWAustralia

**Keywords:** Adverse events, CSL, febrile seizures, fever, influenza vaccine, safety

## Abstract

Influenza vaccine safety is an ongoing issue. In 2010, inactivated trivalent influenza vaccines (TIVs), Fluvax^®^ and Fluvax Junior^®^ manufactured by CSL Biotherapies (‘CSL’), Parkville, Australia, were associated with a marked increase in febrile seizures (FS) in children <5 years old. Extensive investigations initially failed to identify a root cause. The company's researchers recently published two papers outlining their latest findings. Cytokine responses to TIV were measured in paediatric whole blood assays (WBA); NF‐κB activation was assessed using a HEK293 cell line reporter assay. CSL suggest that the combination of new influenza strains (H1N1 A/California/7/2009 and B/Brisbane/60/2008), increased complexes of viral RNA and lipid in the vaccine, and inherent sensitivities of some children <5 years old caused elevated inflammatory responses resulting in FS. Whilst the papers provide insight into pathogenesis, much remains unclear. The WBA were from only 10 ‘healthy’ children, potentially affecting generalisability of the results and reliability of these *in vitro* tests in assessing future influenza vaccine safety. Increased fever rates (without FS) found in CSL TIV studies between 2005 and 2010 suggest a long‐standing contribution to reactogenicity from the manufacturing process. More detailed comparisons with non‐CSL vaccines would have helped elucidate the relative contribution of patient/strain factors and the manufacturing process. The focus remains on manufacturing process differences as the key causative factor of elevated febrile responses. Studies underway, of modified vaccines in young children, will determine whether reactogenicity issues have been successfully addressed and whether CSL TIV can be relicensed in children <5 years of age.

It is extremely important for developed countries to have a viable and responsive local vaccine production capability, for seasonal purposes and especially to deal with the urgent need for a new influenza vaccine with any future pandemic. CSL have fulfilled this role as the major supplier of influenza vaccine in Australia for more than 50 years. However, episodic vaccine safety concerns have the potential to suddenly interrupt vaccine supply from a particular manufacturer and highlight the importance of having multiple vaccine manufacturers in the market.^1^, ^2^

One recent significant safety issue to affect inactivated trivalent influenza vaccine (TIV) involved unforeseen severe febrile reactions and febrile seizures (FS) after vaccination of young Australian children <5 years of age with 2010 Southern Hemisphere (SH) TIV containing the following strains: pandemic H1N1 A/California/7/2009, H3N2 A/Wisconsin/15/2009 and Victoria‐lineage B/Brisbane/60/2008.^3^ This led to the abrupt suspension of the use of seasonal TIV in children aged 5 years and under, on 23 April 2010 whilst the cause was investigated. Therapeutic Goods Administration (TGA) investigations documented 99 FS causally related to seasonal influenza vaccination.^4^ In all 66 cases where the brand of vaccine was recorded, CSL's Fluvax^®^ or Fluvax Junior^®^ was used. Epidemiological analyses revealed a rate of 5–7 FS per 1000 doses of vaccine administered.^4^ Subsequently from 2011, the TGA did not grant approval for Fluvax^®^ use in children aged under 5 years in Australia, and similarly, the US Centers for Disease Control's (CDC) Advisory Committee on Immunization Practices (ACIP) recommended against the use of CSL's product [marketed as Afluria^®^ (Parkville, Australia) in the USA] commencing from the 2010 to 2011 season.^5^ Both organisations also recommended non‐CSL TIV be used in children 5–9 years (Australia) or 5–8 years (USA) unless no alternative was available.^5^, ^6^

The biological basis of increased FS in young children given CSL TIV, however, remained elusive. The TGA undertook a comprehensive laboratory investigation of retention and field samples of CSL's vaccines, guided by a special panel of experts and in conjunction with other regulatory agencies and laboratories in Australia and around the world.^4^ These included assessments of (i) haemagglutinin content by immunodiffusion assay, (ii) presence of bacterial endotoxin, (iii) contamination or differences in chemical profile by chromatographic profiling, (iv) protein characterisation by size exclusion high‐performance liquid chromatography, (v) presence of viral particles or viable virus by transmission electron microscopy and cell culture, (vi) cytokine expression by *in vitro* stimulation testing of peripheral blood mononuclear cells (PBMCs), (vii) presence of RNA and (viii) *in vivo* pyrogenicity using animal models. The analysis found no abnormalities in potency, and no evidence of whole virus particles, viable virus or contamination with endotoxin as possible pyrogenic sources.^4^ Differences were found in the production of a potentially pyrogenic cytokine, TNF‐α, from both CSL's 2009 and 2010 TIV compared with other manufacturers by PBMCs from one donor. If and how this finding was related to the increased FS remained unclear. CSL's manufacturing facilities were also subjected to a Good Manufacturing Process (GMP) audit. Minor inconsistencies were found, which did not present an increased risk to the quality, safety and efficacy of CSL's vaccines.^4^

It became apparent from independent studies that the increased reactogenicity was restricted to recipients of CSL brand vaccines. A retrospective cohort study of Western Australian (WA) children aged <5 years who received TIV in 2010, including CSL's Fluvax^®^/Fluvax Junior^®^, or Influvac^®^ (Solvay Pharmaceuticals, Pymble, Australia),^7^ found that CSL preparations were associated with 62 FS among 14 096 administered doses (a rate of 4·4/1000 doses, 95%CI 3·4–5·6) compared to no FS with 4720 doses of Influvac^®^ (*P* < 0·0001). No comparable peak of FS temporally associated with TIV was seen in the 2008 or 2009 WA paediatric vaccination programmes.^7^ Researchers in New Zealand found three FS with 865 doses of Fluvax^®^ (3·5/1000 doses), but none with 3223 vaccine doses made by other manufacturers.^8^

A laboratory‐based study, reported in 2011, found significant elevations in pyrogenic cytokines and chemokines (IFN‐α, IL‐1β, IL‐6, IL‐10, IP‐10 and MIP‐1α) stimulated by exposure of *ex vivo* PBMCs to 2010 Fluvax^®^ compared with the alternate brands Influvac^®^ (Solvay SA, Brussels, Belgium) or Vaxigrip^®^ (Sanofi Pasteur, Lyon, France).^9^ This study provided an important clue as to the potential mechanism of increased reactogenicity. The subjects were healthy donors (without a specific history of FS) who were age‐matched to children who had presented with febrile adverse events following TIV. The surprising finding of a similar cytokine/chemokine response to the 2009 formulation of Fluvax^®^ suggested a missing piece of the puzzle remained. The 2009 Fluvax^®^ TIV was not temporally associated with increased FS (as measured by ICD‐10 coding) in a West Australian study,^7^ during a year when Fluvax^®^ was known to account for 50–85% of all doses of TIV administered to children in WA.^10^

CSL has been active in attempting to determine the root cause of the events in 2010. Initial studies using various animal models (rabbits, ferrets, newborn rats and rhesus non‐human primates) and *in vitro* stimulation assays using human whole blood following exposure to various TIVs (CSL's 2010 and previous years’ TIVs, re‐engineered 2010 CSL TIV, comparator TIVs) had failed to formally identify a root cause by 2012.^11^ The animal models did not demonstrate a fever response to tested vaccines. However, primate studies provided some clues including elevated serum cytokines in response to the 2010 SH TIV, and changes in gene signature profiles (for multiple immunomodulatory and pro‐inflammatory pathways) in primate blood after exposure to any CSL TIV, especially the 2010 SH TIV.^11^

Rockman *et al*.^3^, ^12^ reported in 2014 on CSL's latest findings. Their latest studies used whole blood assays (WBA) from 19 paediatric donors aged 1–9 years to assess the levels of induced cytokines/chemokines, as well as an *in vitro* human embryonic kidney (HEK293) cell line reporter assay which assessed the level of NF‐κB activation (a master regulator of pro‐inflammatory cytokine responses) after the addition of CSL and comparator TIVs. Their main conclusions about the WBA were derived from 10 of 19 donors aged <5 years old. They found that the 2010 SH CSL TIV, but not previous CSL TIVs, induced elevated cytokines/chemokines in about 30% of tested children, suggesting ‘inherent sensitivities of some paediatric donors (aged <5 years), but not others, to components within the CSL 2010 SH TIV’. The HEK293 cell line reporter assay showed high responses to the 2009/2010 Northern Hemisphere (NH), 2010 SH and 2010/2011 NH CSL TIVs, all of which contained the B/Brisbane/60/2008 strain, relative to either (i) other CSL TIVs without B/Brisbane/60/2008 or (ii) a comparator non‐CSL 2010 TIV. The fact that the highest *in vitro* measurements of NF‐κB signalling were seen with the 2009/2010 NH TIV but FS were only associated with the 2010 SH TIV (and not with the CSL 2009/2010 NH TIV), raises the question whether this assay can reliably predict if any particular future CSL influenza vaccines will provoke FS in children. However, it is unclear how much CSL 2009/2010 NH TIV (marketed as Afluria^®^) was actually used – it was approved by the US Food and Drug Administration only late in that season (10 November 2009). No FS are listed in the US Vaccine Adverse Event Reporting System (VAERS) database for Afluria^®^ in the 2009–2010 season.^13^

Based on the above data on TIVs containing B/Brisbane/60/2008, and the fact that an alternative re‐engineered 2010 SH TIV that replaced only the H1N1 A/California/7/2009 strain with H1N1 A/Brisbane/59/2007 (without any other manufacturing process change), produced lower cytokine/chemokine levels in the WBA, CSL assert that the combination of the B/Brisbane/60/2008 and H1N1 A/California/7/2009 vaccine strains found in the 2010 SH TIV caused a much stronger immune response and increased the risk of FS in a proportion of children aged <5 years. CSL points to three young donors whose WBAs were high responding as evidence to support their assertion. Clearly, these data are limited and involve a *post hoc* analysis regarding a potential predisposition in children who were healthy and lacked a history of FS.

Setting aside for a moment the risk of FS, increased fever‐associated reactogenicity had been associated with CSL TIVs predating the 2010 SH TIV, and we contend that CSL's manufacturing process is the issue of primary importance, even though the new influenza strains may have contributed. CSL themselves somewhat concur, stating ‘CSL's TIVs, as a class were more potent inducers of cytokines *in‐vitro*, as compared to Comparator TIVs’.^3^ They demonstrated that the cytokine/chemokine profile for CSL 2010 SH TIV was highly significantly elevated compared to the two unnamed comparator vaccines (2010 SH and 2010/2011 NH) but seemingly not significantly different to CSL 2009 SH TIV, containing the Yamagata‐lineage B/Florida/4/2006 strain (also found by CSL to potentially cause an elevated cytokine response).^3^ This suggests that a number of CSL TIVs have been associated with an increased inflammatory response resulting in fever.

It seems remiss that the authors, whilst presenting levels of cytokines induced by several CSL TIVs and individual influenza strains, presented only limited comparison to the aforementioned non‐CSL comparator vaccines. In particular, we were not shown cytokine levels for comparator vaccines tested in their three high‐ and seven low‐responding paediatric donors.^3^ This would have helped put the CSL vaccines’ results in the context of other manufacturers’ vaccines, and allowed better interpretation of the relative contributions of the strain and patient factors compared with manufacturing factors in causing the elevated cytokine/chemokine levels. Also, one cannot be sure that the 3 ‘high responders’ were representatives of the group genetically prone to increased reactogenicity. It would be helpful to see WBAs carried out on young children who actually suffered FS, in association with TIV as well as other triggers.

It has been argued that the increases in FS seen in 2010 ‘could not have been predicted from either the clinical trial data or the post marketing reports generated in the paediatric population prior to the 2010 SH season’.^3^ However, CSL's previous paediatric studies with their own vaccines, produced before the 2010 SH TIV, whilst not being associated with a high level of FS, showed concerning levels of fever, a necessary precursor of FS. Two uncontrolled trials conducted in 2005–2006 and 2009 documented fever rates in children aged 6–35 months of up to 39·5% and 28·6%, respectively.^14^, ^15^ These studies also documented FS, but only one in each study, both occurring on the day of vaccination among 197^14^ and 710^15^ vaccinees, respectively. Neither study involved vaccines containing B/Brisbane/60/2008 or H1N1 A/California/7/2009 strains. B/Florida/4/2006 strain, which induced raised cytokine levels but to a lesser degree,^3^ was used in one study.^15^

Importantly, our own systematic review of post‐TIV fever rates^16^ for the 6‐ to 35‐month age group in randomised controlled trials from multiple manufacturers, and several other observational studies and reviews^17^, ^18^, ^19^ have established that typical rates of fever after non‐CSL TIVs are approximately 6–8% in this age group. Of additional concern, a RCT conducted in 2009 in the USA and recently published in 2014^20^ compared CSL's Afluria^®^ with a Sanofi comparator (Fluzone^®^) and documented significantly more fever after the first dose of Afluria^®^ in children aged 6–35 months (37·1% versus 13·6%, respectively, *P* < 0·0001) and in those aged 3–8 years (21·8% versus 9·4%, respectively, *P* = 0·0001). This CSL vaccine did contain B/Brisbane/60/2008, plus A/Brisbane/59/2007 (H1N1) and A/Uruguay/716/2007 (H3N2). Unfortunately, these data were not available prior to the events of 2010 in Australia.

The excessive febrile reactions with 2010 Fluvax^®^ were independently suspected,^21^ and subsequently confirmed by CSL,^12^ to relate to their method of manufacture. Their recent analysis using the NF‐κB HEK293 reporter assay elaborated on this mechanism: fragments of viral RNA retained during TIV manufacture, which then required viral lipid‐mediated delivery to the cytoplasm of host antigen‐presenting cells. CSL have found that increases in the concentration of sodium taurodeoxycholate (TDOC), the splitting agent used in the production of Fluvax^®^, from 0·5% to 1·5% for B strains, substantially reduced the level of viral RNA and lipid.^12^ Currently, CSL is alone in using TDOC as a splitting agent, although GlaxoSmithKline use sodium deoxycholate for Flulaval^®^ and Fluarix^®^.^22^ CSL are investigating with a current clinical trial whether the change in TDOC concentration reduces the reactogenicity and the risk of FS with their vaccines.^23^ As there can be a fine balance between reactogenicity and immunogenicity, it will be of interest to know whether changes to CSL's manufacturing process aimed at reducing fever and the risk of FS has any impact on immunogenicity of their vaccines in children.

Ultimately, the most important limitation in CSL's recent papers is that all data presented come from *in vitro* studies using donor whole blood or a HEK293 NF‐κB reporter assay.^3^, ^12^ There is no *in vivo* predictive test. The human *ex vivo* WBA data on young children are based on just ten donors.^3^ Whilst the cytokine levels in high‐responding donors' WBA did show some difference between CSL's 2010 TIV compared to the previous and subsequent years' TIVs, the profile for all donors' samples combined did not appear markedly different between years (Figure 2 of Ref. 3). In addition, it is evident from this figure that high‐responding individuals appear to vary between the various cytokine/chemokine assays for different TIVs. This raises the issue of reproducibility for this assay. If CSL uses this assay to assist with identification of potential future reactogenic vaccine formulations, how will they be certain about the appropriate safety threshold? Validation of CSL's two *in vitro* tests by examining children who had previously suffered fever or FS after CSL TIV, or who are recorded to have such events in future CSL clinical trials of its TIVs could prove very valuable.

Additional information is required outlining clinical factors that may predispose to a febrile reaction after influenza vaccine to inform future safety studies. One option to assist with this would be by allowing public access to individual‐level clinical trial data from the past and present paediatric TIV studies for independent analysis, as is currently offered to varying degrees by other vaccine manufacturers (GlaxoSmithKline, Brentford, England; Novartis, Basel, Switzerland; Sanofi, Paris, France)^24^ but which is not currently available for CSL studies. This would allow more precise characterisation of the fever profile after CSL TIVs, and better identification of patients predisposed to febrile adverse events. It would provide a more substantial basis to test, in those most at risk, if immune activation with new formulations is still an issue.

The recent acquisition by CSL of Novartis's global influenza vaccine business will create the second largest vaccine company in the global influenza industry.^25^ In the light of CSL's plans to continue the clinical development of its own Australian‐made influenza vaccine alongside that of Novartis's vaccines, establishing whether the safety concerns arising from the events of 2010 have been successfully addressed remains important. It is still uncertain whether proposed changes in manufacturing methods by CSL will resolve the issues of increased reactogenicity with their TIVs in young children and bring them in line with comparators. The development of a quadrivalent product will complicate this. Access to new technology acquired from Novartis's influenza vaccine business may assist CSL in the development of safer influenza vaccines for children. Currently, a safety study with reformulated vaccine given to 5‐ to 9‐year olds is underway and will hopefully provide some answers.^23^ Subsequent studies in younger children, with rigorous scrutiny and prompt reporting of results, are needed before consideration can be given to relicensing CSL TIVs in children under 5 years of age.

## Conflict of interests

R. Booy has received funding from bioCSL, Roche, Abbott, Sanofi, GlaxoSmithKline (GSK), Novartis and Pfizer to conduct sponsored research or attend and present at scientific meetings, or produce educational material by employing a researcher; any funding received is directed to a research account at the Children's Hospital at Westmead.
